# The paradox of higher light tolerance during desiccation in rare old forest cyanolichens than in more widespread co-occurring chloro- and cephalolichens

**DOI:** 10.1111/j.1469-8137.2012.04221.x

**Published:** 2012-09

**Authors:** Yngvar Gauslaa, Darwyn S Coxson, Knut Asbjørn Solhaug

**Affiliations:** Department of Ecology and Natural Resource Management, Norwegian University of Life SciencesPO Box 5003, NO-1432 Ås, Norway

**Keywords:** cephalolichens, chlorolichens, cyanolichens, desiccation tolerance, epiphytes, hydration regime, light tolerance, *Nostoc*

## Abstract

- Desiccation tolerance was quantified in four cyanolichens (*Lobaria hallii*, *Lobaria retigera*, *Lobaria scrobiculata*, *Pseudocyphellaria anomala*), one cephalolichen (*Lobaria pulmonaria*) and one chlorolichen (*Platismatia glauca*) from xeric and mesic, open and closed North American boreal forests.
- These sympatric epiphytes were exposed to 0%, 33%, 55% and 75% relative humidity with or without medium light (200 μmol m^−2^ s^−1^) for 7 d. Permanent and temporary photoinhibitory damage was recorded as viability measures.
- All species tolerated well the drying in darkness, but *L. hallii* and *L. retigera*, associated with a very humid climate, showed minor damage at the hardest drying (silica gel). Simultaneous exposure to medium light severely aggravated the drying damage at all relative humidity levels. Combined drying–light exposure was particularly devastating for the widespread chloro- and cephalolichens, whereas cyanolichens, including rare old forest species, were fairly resistant.
- The ability to recover after combined drying–light stress (this study) correlated positively with increasing species-specific water holding capacities (from the literature). Cyanolichens, depending on liquid water and large internal water storage, probably require strong drying–light resistance to handle long periods between hydration events, whereas chlorolichens can regularly maintain their photosynthetic apparatus during frequent and rapid activation by humid air on clear mornings.

## Introduction

Lichens are poikilohydric symbiotic associations between a mycobiont and one or more photobionts (green algae and/or cyanobacteria). Some lichens are extremely resistant to drying and extreme UV radiation, being alive after several days in outer space (Sancho *et al.*, 2007; de la Torre *et al.*, 2010). This tolerance is remarkable, as drying switches off dark reactions of photosynthesis, allowing the accumulation of harmful oxygen species (reactive oxygen species, ROS) when exposed to light (Kranner *et al.*, 2008). However, drying activates special energy-dissipating mechanisms in lichens (Heber *et al.*, 2006). Lichens with green algal photobionts using the energy-dissipating xanthophyll cycle, as well as cyanolichens lacking this cycle, both show strong drying-induced quenching of chlorophyll fluorescence (Heber *et al.*, 2006). Thereby, both photobiont groups use a photosystem II (PSII) reaction center mechanism to dissipate energy when dry (Heber *et al.*, 2006, 2010). The efficiency of protection mechanisms varies among lichens. Old forest cephalolichens (= chlorolichens with cyanobacteria in localized colonies, called cephalodia) are susceptible to high-light exposure in the dry state (Gauslaa & Solhaug, 1996).

Most members of the lichenized fungal order Peltigerales live in forests, particularly species in the two related genera *Lobaria* and *Pseudocyphellaria* treated in this article. They include cephalolichens as well as cyanolichens. Unlike other taxonomic groups, green algal and cyanobacterial members of Peltigerales consistently produce extracellular superoxide radicals at high rates after drying (Beckett *et al.*, 2003). As the vast majority of these lichens are restricted to forests and/or humid climates (Marini *et al.*, 2011), their distribution patterns may be shaped by intolerance to prolonged desiccation. At the same time, a number of cephalolichens in the Peltigerales are more adversely affected by high light when dry than are pure chlorolichens of other taxonomic groups (e.g. the Parmeliaceae) without cephalodia (Gauslaa & Solhaug, 1996, 1999, 2004). So far, photoinhibition in dry cyanolichens has not been studied in detail. The classical photoinhibition study of Demmig-Adams *et al.* (1990b) showed that cyanolichens had a lower recovery than chlorolichens after short-term high-light exposures whilst hydrated. They related the higher susceptibility of cyanolichens to the absence of xanthophyll cycle pigments in cyanobacteria (Demmig-Adams *et al.*, 1990a). Cyanolichens that had dried in light before short-term high-light exposures exhibited greatly accelerated recovery (Demmig-Adams *et al.*, 1990c). Similar responses after drying in light were seen in the cephalolichen *Lobaria pulmonaria* (Štepigová*et al.*, 2008). However, during clear weather, high-light exposure sometimes lasts for many days without hydration, even at night. Lichens thus experience much longer periods of high light when dry than when hydrated. During light treatments in extended dry periods, irreversible damage accumulates with time (Gauslaa & Solhaug, 1996, 1999). As some cyanolichens tend to dominate the wettest forests and canopies in the spray zone of waterfalls (Goward & Spribille, 2005), their rareness and absence in drier sites are often ascribed to low drying tolerance, although experiments separating the effects of drying from those of simultaneous light exposure are lacking and their drying tolerance is unknown.

Water loss and uptake in lichens are physical processes. Their water potential reflects the water potential in the air. Lichens frequently experience water potentials too low for photosynthesis. Chloro- and cephalolichens have positive photosynthesis in the absence of liquid water at 97% relative humidity (RH) with a water potential (*Ψ*_air_) of *c*. − 4 MPa, whereas cyanolichens require liquid water to activate photosynthesis (Lange *et al.*, 1986). Positive CO_2_ exchange in two chlorolichens continued down to − 22 and − 38 MPa (Nash *et al.*, 1990), whereas no further photosynthesis occurred in four other chlorolichens at − 40 MPa (Scheidegger *et al.*, 1995). In drying-tolerant bryophytes, little or no photosynthesis occurred below – 15 to 20 MPa (Dilks & Proctor, 1979). However, metabolic processes other than photosynthesis take place at low rates down to at least 50% RH in cyano- and chlorolichens (Cowan *et al.*, 1979). As our study aims to quantify drying damage, we study the effects of water potentials below the threshold for photosynthetic activity.

This study focuses on six sympatric foliose epiphytic lichens being locally dominant on conifer branches and stems of deciduous trees in old forests and/or humid boreal climates. By comparing photoinhibition in a mix of co-occurring cyano-, chloro- and cephalolichens in experiments using fixed RHs with and without light exposure, we aim to identify possible photobiont-specific responses to drying. Earlier studies of drying susceptibility have been conducted in darkness or light (e.g. Green *et al.*, 1991; Kranner *et al.*, 2003), but the light factor has rarely been considered to be relevant. We aim to discriminate between damage caused by drying alone and by combined light and drying exposure. Lichen anatomy, specific thallus mass and water holding capacity (WHC) have already been quantified in all of our specimens by Gauslaa & Coxson (2011). In order to understand lichen functioning, we intend to analyze whether inter- and/or intraspecific differences in these internal variables can explain parts of the variation in drying and/or light susceptibility assessed in this study. Finally, we wish to analyze possible implications of the results for lichen physiology, distribution patterns and rareness. As members of *Lobaria* and *Pseudocyphellaria* are species of concern, indicating ecological continuity in forest conservation management (Rose, 1976), knowledge on the basic responses to drought and high-light stress may facilitate ecologically viable management plans to secure declining and species-rich epiphytic communities in our forests.

## Materials and Methods

### Lichen material

We included chlorolichens (*Platismatia glauca* (L.) W.L.Culb. & C.F.Culb. with *Trebouxia* as the photobiont), cephalolichens (*Lobaria pulmonaria* (L.) Hoffm. with the green alga *Dictyochloropsis* as the main photobiont and the cyanobacterium *Nostoc* in cephalodia) and cyanolichens (with *Nostoc* only; *Lobaria hallii* (Tuck.) Zahlbr., *L. retigera* (Bory) Trevisan, *L. scrobiculata* (Scop.) DC., *Pseudocyphellaria anomala* Brodo & Ahti). The most widespread lichen, *P. glauca*, belongs to the Parmeliaceae, whereas the other species are members of the Lobariaceae in the Peltigerales. *Lobaria hallii* is mainly a species of spray zones of waterfalls in Europe (Jørgensen & Tønsberg, 2007) and humid forests west of the Rocky Mountains in North America (Goward *et al.*, 1994), whereas *L. retigera* grows in very humid forests of western North America and eastern Asia. In subtropical–temperate moist forests in New Zealand, *L. retigera* is one of few lichens that, together with the highly drying-susceptible *Pseudocyphellaria dissimilis* (Green *et al.*, 1991), successfully competes with bryophytes in very low light on damp forest floors (Galloway, 1988).

All six species were collected along a longitudinal gradient in British Columbia in April 2010 (Table 1). The three easternmost sites (Slim, Upper Fraser and Aleza) are located within or at the edge of mountain valleys, whereas the two westernmost sites fall within the drier interior plateau region of central-interior British Columbia. Although not all species were collected at each site (Table 1), these are co-occurring species throughout this region, although their abundance varies between sites (see Doering & Coxson, 2010). The exception to this pattern of co-occurrence was *L. retigera*, which was only found in the Slim Creek site (Table 1). These sites, from east to west, respectively, fall within the following biogeoclimatic subzones (from Meidinger & Pojar, 1991): Slim Creek – the very wet cool interior cedar–hemlock subzone; Upper Fraser and Aleza – the wet cool sub-boreal spruce climate subzone; and Salmon Valley and Prince George – the moist cool sub-boreal spruce climate subzone. The mean annual temperature in these sites varies from 2.6 to 3.5°C, with the two westernmost sites, Salmon River and Prince George, being markedly drier. Lichens collected (Table 1) from open-canopy sites (Upper Fraser and Salmon River) were from *Salix discolor* in mixed riparian woodlands with *Alnus incana*, *Populus tremuloides* and *P. balsamifera.* The mean canopy openness during the growing season in these riparian sites was 11%, with 980 stems ha^−1^ over 10 cm in diameter at breast height (Doering & Coxson, 2010). The Slim Creek closed-canopy site was dominated by *Thuja plicata* and *Tsuga heterophylla*. Radies *et al.* (2009) found that the mean canopy openness in wet interior (cedar–hemlock zone) coniferous stands in the Slim Creek area was 14.1%. Closed-canopy forests at both Aleza and Prince George were dominated by hybrid spruce, *Picea engelmannii × glauca* and *Abies lasiocarpa*. Campbell *et al.* (2010) found that the average light transmission at Aleza was 21.2% (direct plus diffuse light expressed as a percentage of the maximum).

**Table 1:** Climate variables^1^ and location for lichen collection sites in British Columbia, Canada

Site	Type	Location (latitude/longitude)	Elevation (m)	Mean annual precipitation (precipitation as snow; mm)	Mean annual temperature (mean July–August temperature, °C)	Collected lichens
Prince George	Xeric open (plateau)	53°53′07.12′′N 122°49′13.69′′W	785	570 (205)	3.5 (14.2)	*P. glauca*
Salmon River	Xeric open (plateau)	54°12′22.43′′N 122°41′48.83′′W	743	668 (279)	2.6 (13.4)	*L. hallii*, *L. pulmonaria*, *L. scrobiculata*, *P. anomala*
Aleza	Mesic closed (mountain)	54°05′55.29′′N 122°05′02.84′′W	660	888 (350)	3.0 (14.3)	*L. hallii*, *L. pulmonaria*, *L. scrobiculata*, *P. anomala*, *P. glauca*
Upper Fraser	Mesic open (mountain)	54°05′23.14′′N 121°52′07.78′′W	608	816 (313)	3.2 (14.4)	*L. hallii*, *L. pulmonaria*, *L. scrobiculata*
Slim Creek	Mesic closed (mountain)	53°45′24.20′′N 121°12′05.25′′W	778	838 (315)	3.0 (13.4)	*L. retigera*

1 Temperature and precipitation normals for 1961 to 1990 from Climate BC modelling (Wang *et al.*, 2006); see http://genetics.forestry.ubc.ca/cfgc/ClimateBC/Default.aspx.

To develop experimental protocols for this study, we performed a number of pre-experiments with Norwegian material of *L. pulmonaria* sampled in *Picea abies*-dominated forests at Namsos, central western Norway (64°20–25′N, 11°16–30′E) from the same collection as used by Larsson *et al.* (2012). The Norwegian thalli responded exactly like those from British Columbia with respect to light and desiccation treatments.

With the large number of sites, species, treatments and replicates, we used thallus disks to obtain enough space under the experimental set-up. Lichens were hydrated by spraying de-ionized water on the upper surface in the laboratory. From each moistened thallus, one or two disks with an area of *c*. 1 cm^2^ each were taken at random positions by a cork borer. A similar number of disks (*n* = 40) were taken from each species in each of the collection sites (Table 1), amounting to 560 disks in total. The disks were randomly selected species-wise for each treatment. All disks were preconditioned to allow relaxation of the short-term down-regulation of PSII. This was performed by spraying the disks with de-ionized water on the upper surface and keeping them hydrated in low light (10 μmol photons m^−2^ s^−1^) at room temperature (20°C) for 48 h. Immediately after the preconditioning, *F*_v_/*F*_m_ was recorded with a portable fluorimeter (Plant Efficiency Analyser; Hansatech, King’s Lynn, Norfolk, UK) after a 15-min period of dark adaptation. The disks were then dried until the next day between filter papers, using gentle pressure to keep them dry and flat before the drying and light treatments commenced. Treatments were conducted in clear 250-ml plastic boxes (10 × 8 × 4 cm^3^; length, width, height). The experiment was replicated three times. In each replication, disks for each species were randomly selected. Within each replicated experiment, disks were also randomized for each box. Both the measured *F*_v_/*F*_m_ values and *F*_v_/*F*_m_ as a percentage of initial preconditioning values are presented. This recognizes the generally lower *F*_v_/*F*_m_ values in healthy cyanolichens relative to healthy cephalo- and chlorolichens. Only the last two groups with green algae have *F*_v_/*F*_m_ values as high as those of vascular plants (for a further discussion of these differences, see Lüttge, 2011).

### Drying treatments

Air-dry lichen disks were placed on a net fastened 5 mm below the top of each box. Relative humidities of 0%, 33%, 55% and 75% were obtained by placing silica gel and saturated solutions of MnCl_2_, Mn(NO_3_)_2_ and NaCl, respectively (Greenspan, 1977) in the bottom of each box. The boxes were tightly covered with cling film. Four boxes were used for each humidity level, two for each light treatment. Drying lasted for 7 d. Five additional disks were used for water content measurements at each humidity level for both light treatments. After 7 d of treatment at constant RH levels, these lichen disks were immediately weighed with the absolute dry mass subsequently being determined after drying at 70°C for 24 h. All studied lichen species had fairly equal water contents after equilibrium with each relative air humidity level, but *P. glauca* had slightly higher water contents than the other species (Table 2). The water content of lichens varied from 3.8% to 6% in 0% RH and from 13.7% to 16.3% in 75% RH. This range in RH and corresponding lichen water content spans more or less the entire humidity gradient below which photosynthetic activity in lichens can start. The water potential in the air (*Ψ*_air_) was calculated using the equation:


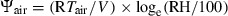
Eqn 1

**Table 2:** Mean percentage water content in lichen thalli above silica gel and various saturated salt solutions after 7 d under 200 μmol m^−2^ s^−1^

RH (%)	Silica or salt solution	*Ψ*_air_, MPa	*Lobaria hallii*	*Lobaria pulmonaria*	*Lobaria retigera*	*Lobaria scrobiculata*	*Platismatia glauca*	*Pseudocyphellaria anomala*
0	Silica gel	−∞	4.2 ± 0.6	3.8 ± 0.6	5.1 ± 1.2	3.8 ± 0.7	6.1 ± 0.5	4.2 ± 0.6
35	MgCl_2_	−148	8.5 ± 0.1	8.6 ± 1.1	8.1 ± 0.1	7.8 ± 0.2	9.9 ± 0.2	9.5 ± 1.2
55	Mg(NO_3_)_2_	−80	11.4 ± 0.4	10.8 ± 0.2	12.1 ± 0.7	10.8 ± 0.1	12.3 ± 0.4	11.4 ± 0.4
75	NaCl	−38	15.8 ± 0.1	14.3 ± 0.2	15.4 ± 0.1	14.4 ± 0.6	16.3 ± 1.31	13.7 ± 0.6

RH, relative humidity. Values are mean ± 1SE; *n* = 5.

(R, gas constant (8.31441 J mol K^−1^); *T*, air temperature assumed to be the same as the salt solution temperature; *V*, partial molar volume of water (18.0 × 10^−6^ m^3^ mol^−1^)) (Jonsson *et al.*, 2008).

### Light treatments

**Fig. 1:**
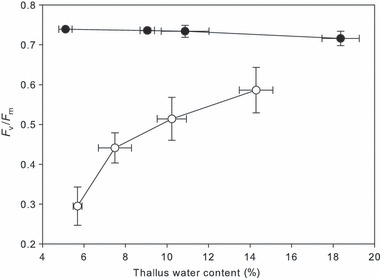
Maximum quantum yield of photosystem II (*F*_v_/*F*_m_; measured in hydrated thalli of Norwegian *Lobaria pulmonaria* after 48 h of recovery subsequent to drying treatment) as a function of the percentage water content in thalli experiencing 7 d of drying in darkness (closed circles) or at 200 μmol m^−2^ s^−1^ (open circles). Drying treatments were given in boxes in which thalli were in equilibrium with 0% (the lowest water content), 35%, 55% and 75% relative humidity. Error bars show standard errors.

During the entire drying experiment, 50% of the boxes with lichen disks were exposed to medium light (200 μmol m^−2^ s^−1^) from a high-intensity light-emitting diode (LED) panel (LED light source, model SL3500; Photon Systems Instruments, Brno, Czech Republic). The light treatment was *c.* 10% of the maximal irradiance experienced under boreal field conditions (Gauslaa & Solhaug, 2000) and 200 μmol m^−2^ s^−1^, which is close to the light saturation point for photosynthesis in *L. pulmonaria* and *L. hallii* from our localities (K. A. Solhaug & X. Lie, unpublished), and is frequently encountered in lichen sites of the studied forest type in British Columbia (Coxson & Stevenson, 2007a). The light source had blue, green and red diodes which were individually regulated to give equal irradiance. The light exposure experiment was performed in a temperature-regulated chamber at 14°C. This gave a thallus temperature of 20°C (measured with a thin thermocouple) in closed boxes; the temperature in the shaded salt solutions beneath the lichens was *c*. 16°C. The boxes with the other 50% of lichen disks above the same salt solutions were kept in continuous darkness for the same period of time. However, they were kept at 20°C to reduce differences in temperature between the light and dark treatments. The light slightly reduced the water content at the three highest RH levels, but not in those kept over silica gel, as shown by an experiment on the Norwegian *L. pulmonaria* thalli (Fig. 1) as part of testing and developing the experimental set-up. On average, for all RH levels, the lichen water content was 1.5% higher in the dark controls.

After the treatments, disks were taken out of the boxes, placed on moist filter papers and given the same conditions during the 48-h recovery as during preconditioning. *F*_v_/*F*_m_ (see earlier) was repeatedly measured during the recovery period.

### Statistical analyses

One-way ANOVAs including the Holm–Sidak method (as given in Table 3) and linear regression models (Fig. 4) were computed in SigmaPlot 11.0 (Systat Software Inc., San Jose, CA). Two-way ANOVAs (Table 4) and repeated measures three-way ANOVAs (general linear model; as given in Table 5) were performed in Minitab 16 (Minitab Inc., State College, PA).

**Table 3:** Habitat-specific mean *F*_v_/*F*_m_ values before and after the start of the drying experiment for specimens selected for darkness and light treatment (200 μmol m^−2^ s^−1^)

	*F*_v_/*F*_m_Before start of experiment		*F*_v_/*F*_m_After drying and 48 h recovery		*F*_v_/*F*_m_Percentage of start values	
			
	Darkness	Light	Darkness	Light	Darkness	Light
*Lobaria pulmonaria*						
Xeric open	0.684 ± 0.010a	0.680 ± 0.015a	0.724 ± 0.007a	0.528 ± 0.056a	106.2 ± 1.2b	77.5 ± 8.1b
Mesic open	0.702 ± 0.011a	0.700 ± 0.012a	0.725 ± 0.004a	0.533 ± 0.054a	103.7 ± 1.5b	77.2 ± 8.0b
Mesic closed	0.736 ± 0.002b	0.733 ± 0.003b	0.733 ± 0.003a	0.431 ± 0.045a	99.6 ± 0.3a	58.8 ± 6.1a
*Platismatia glauca*						
Xeric open	0.691 ± 0.013b	0.686 ± 0.019a	0.721 ± 0.012b	0.538 ± 0.040b	104.5 ± 1.1a	79.3 ± 5.6b
Mesic closed	0.612 ± 0.020a	0.651 ± 0.017a	0.655 ± 0.018a	0.270 ± 0.053a	108.0 ± 2.6a	40.3 ± 7.7a
*Lobaria hallii*						
Xeric open	0.524 ± 0.007ab	0.520 ± 0.009a	0.488 ± 0.010a	0.438 ± 0.011a	93.2 ± 1.4a	84.2 ± 1.1a
Mesic open	0.510 ± 0.006a	0.491 ± 0.009a	0.474 ± 0.008a	0.422 ± 0.008a	92.9 ± 1.0a	86.2 ± 1.8a
Mesic closed	0.543 ± 0.008b	0.546 ± 0.009b	0.503 ± 0.010a	0.457 ± 0.014a	93.0 ± 1.0a	83.6 ± 1.7a
*Lobaria retigera*						
Mesic closed	0.518 ± 0.006	0.520 ± 0.005	0.494 ± 0.007	0.429 ± 0.010	95.4 ± 1.3	82.5 ± 2.0
*Lobaria scrobiculata*						
Xeric open	0.521 ± 0.012a	0.530 ± 0.010a	0.547 ± 0.007a	0.521 ± 0.013a	105.7 ± 1.9b	98.7 ± 2.6a
Mesic open	0.542 ± 0.007a	0.551 ± 0.006a	0.549 ± 0.008a	0.506 ± 0.014a	101.4 ± 1.2ab	91.8 ± 2.2a
Mesic closed	0.548 ± 0.007a	0.527 ± 0.010a	0.548 ± 0.007a	0.492 ± 0.014a	100.0 ± 0.7a	93.6 ± 2.4a
*Pseudocyphellaria anomala*						
Xeric open	0.594 ± 0.007a	0.586 ± 0.013a	0.580 ± 0.005a	0.538 ± 0.021a	97.8 ± 0.9a	91.0 ± 2.5a
Mesic closed	0.581 ± 0.005a	0.577 ± 0.004a	0.572 ± 0.006a	0.500 ± 0.014a	98.5 ± 0.9a	86.7 ± 2.4a

Values are mean ± 1SE; *n* = 19–20.

Means combined measurements from all four relative humidity levels during the 7-d experiment. Within each species sampled at more than one site, an ANOVA was run to test whether the sampled sites differed significantly from each other (*P* < 0.05). Within each species, sites sharing the same letter for a given variable were not statistically different from each other (all pairwise multiple comparison procedures: Holm–Sidak method).

**Table 4:** Two-way ANOVA for the level of photoinhibition after combined drying and light exposure in *Lobaria pulmonaria* and *Platismatia glauca* from different habitats, and exposed to four levels of desiccation (see Fig. 2)

Species	*Lobaria pulmonaria*				*Platismatia glauca*			
				
Source	df	*F*	*P*		df	*F*	*P*	
Relative humidity (RH)	3	35.95	0.000		3	12.25	0.000	
Habitat	2	3.54	0.045		1	54.78	0.000	
RH × habitat	6	3.17	0.020		3	7.32	0.003	
Error	24				16			
Total	35				23			
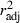				77.5				82.2

Photoinhibition was expressed as *F*_v_/*F*_m_ as a percentage of the start values. Factors were RH drying (0%, 35%, 55% and 75% RH) and habitat types specified in Table 3 for each species.

**Table 5:** Repeated measures three-way ANOVA (general linear model) for *F*_v_/*F*_m_ after drying at four levels with and without light exposure for six species nested in two photobiont types (green algae or cyanobacteria as main photobiont)

Source	df	*F*	*P*
C1 (humidity light photobiont species)	507	6.34	0.000
Time (repeated during recovery process)	4	1332.28	0.000
Humidity	3	71.03	0.000 x
Light	1	1960.80	0.000 x
Species (nested in photobiont)	4	10.39	0.000 x
Photobiont	1	189.13	0.000 x
Time × humidity	12	4.22	0.000
Time × light	4	233.32	0.000
Time × species (photobiont)	16	15.84	0.000
Time × photobiont	4	50.60	0.000
Humidity × light	3	55.36	0.000 x
Humidity × species (photobiont)	12	1.31	0.208 x
Humidity × photobiont	3	9.65	0.000 x
Light × species (photobiont)	4	3.19	0.013 x
Light × photobiont	1	456.97	0.000 x
Time × humidity × light	12	8.90	0.000
Time × humidity × species (photobiont)	48	1.71	0.002
Time × humidity × photobiont	12	17.23	0.000
Time × light × species (photobiont)	16	4.78	0.000
Time × light × photobiont	4	16.18	0.028 x
Humidity × light × species (photobiont)	12	1.94	0.001 x
Humidity × light × photobiont	3	5.77	0.000
Time × humidity × light × species (photobiont)	48	1.14	0.234
Time × humidity × light × photobiont	12	16.05	0.000
Error	2011		
Total	2757		

*F*_v_/*F*_m_ was repeatedly measured during recovery at 0.5, 2, 6, 24 and 48 h after drying (0%, 35%, 55% and 75% relative humidity (RH) and light treatments (0 and 200 μmol m^−2^ s^−1^) lasting for 7 d. Data are shown in Fig. 3. 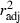 = 91.73%. x, not an exact *F* test.

## Results

In general, habitat type had a low impact on the measured variables in the studied lichens (see Table 3) in which *F*_v_/*F*_m_ values after the drying experiment had been averaged across all four humidity treatments. *F*_v_/*F*_m_ at the start was much more species specific than habitat specific, with the highest values in *L. pulmonaria* followed by *P. glauca*. The cyanolichen with the highest *F*_v_/*F*_m_ values was *P. anomala*, whereas the three cyanobacterial *Lobaria* species had the lowest starting *F*_v_/*F*_m_ values. In *L. pulmonaria* and *L. scrobiculata*, there was a slight tendency to the highest *F*_v_/*F*_m_ value in the most shaded site (Table 3), even after 48 h of preconditioning of hydrated thalli at low light.

After 7 d of desiccation in darkness, followed by moistening and 48 h of recovery at low light, *F*_v_/*F*_m_ was hardly affected. Indeed, *F*_v_/*F*_m_ after drying in darkness, expressed as a percentage of *F*_v_/*F*_m_ before the start, exceeded 100% in the three most widespread species (*P. glauca*, *L. pulmonaria* and *L. scrobiculata*). This showed that the PSII efficiency of these forest lichens was not adversely affected by 1 wk of continuous drying in darkness. Only the two rarest species (*L. hallii* and *L. retigera*), apparently restricted to very humid sites, showed slight, but significant, reductions: 7% and 5%, respectively (Table 3). As this reduction was measured after as much as 48 h of recovery at low light, the reduction can be considered to be permanent slight damage.

**Fig. 2:**
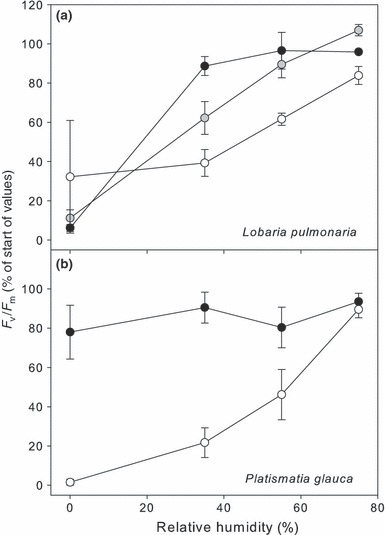
Level of photoinhibition after combined drying (0%, 35%, 55% and 75% relative humidity) and light exposure (200 μmol m^−2^ s^−1^) in *Lobaria pulmonaria* and *Platismatia glauca* from different habitats (black circles, xeric open; gray circles, mesic open; white circles, mesic closed) as specified in Table 3. Each symbol represents the mean ± 1SE. See Table 4 for ANOVA results.

Using the same drying treatments in combination with medium light (200 μmol m^−2^ s^−1^), permanent photoinhibition occurred in all species (Table 3). The chloro- and cephalolichens were clearly the most severely affected, with visible bleached portions in many disks experiencing the hardest drying regimes. In these two functional groups, in which the habitat had the greatest influence, thalli from the closed forests showed the greatest photoinhibition after the combined drying and light exposure (Fig. 2, Table 4). In the cephalolichen (*L. pulmonaria*; Fig. 2a; Table 4), the effect of habitat was weak, but significant, whereas in the chorolichen (*P. glauca*; Fig. 2b; Table 4), the effect of habitat was very strong. *Platismatia glauca* from the open xeric forest was not affected by the level of drying, whereas thalli from the mesic closed forest experienced severely aggravated photoinhibition with decreasing RH (Fig. 2b; Table 4). None of the cyanolichens were as severely affected as those with green algal photobionts. Nevertheless, when drying occurred in combination with light, *L. hallii* and *L. retigera* were the most severely affected among the cyanolichens with > 15% reduction in *F*_v_/*F*_m_. The lowest level of permanent photoinhibition occurred in the most widespread cyanolichen (*L. scrobiculata:* Table 3).

**Fig. 3:**
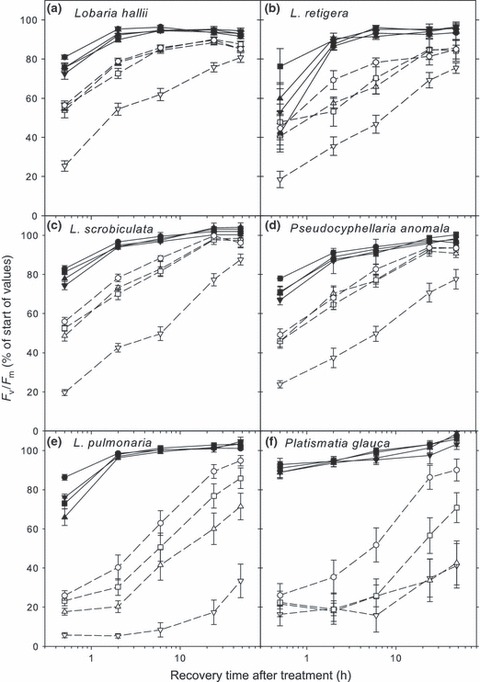
Kinetics during recovery (log-scale) for hydrated thalli of the studied species at low light after 7 d of drying treatment in darkness (closed symbols and solid lines) and in medium light (200 μmol m^−2^ s^−1^; open symbols and dotted lines). Each symbol represents the mean ± 1SE. Relative humidity during drying: triangles apex down, 0%; triangles apex up, 35%; squares, 55%; circles, 75%.

Details on the recovery kinetics in *F*_v_/*F*_m_ after each of the four drying treatments with and without light are shown for all species in Fig. 3. As the influence of habitat was relatively low (Table 3), mean values across all habitats are given in Fig. 3. The dataset in Fig. 3 was analyzed by repeated measures ANOVA, with time as the repeated measurement during recovery, and species nested in the two photobiont groups: green alga and cyanobacteria (Table 5). This analysis suggested that up to 91.7% of the variation in the nearly 2800 recordings of *F*_v_/*F*_m_ could be accounted for by the variables humidity level, light level, species and time. The ANOVA showed that photobiont type better predicted *F*_v_/*F*_m_ than did the species (Table 5).

Recovery was rapid and complete, or nearly complete, in all lichen species subjected to drying in darkness (Fig. 3). For lichens experiencing drying in darkness, the activation time for restoring normal photosynthesis during hydration after drying exposure was shortest in the widespread *P. glauca* (Fig. 3), evidenced by high *F*_v_/*F*_m_ values already after 30 min. The slowest activation of photosynthesis after drying in darkness occurred in the rare and humidity-dependent *L. retigera*. Schlensog *et al.* (2004) made the point that the time to recover is related to the length of active time; lichens that remain active for short times recover most rapidly. Light during drying was the most significant factor for *F*_v_/*F*_m_, with a close to 30 times higher *F* value than that of the humidity factor (Table 5). The light treatment substantially delayed the activation of photosynthetic responses after subsequent wetting, particularly in the two green algal lichens. Light also significantly aggravated the permanent (48 h) photoinhibition caused by drying (Fig. 3).

*F*_v_/*F*_m_ declined with decreasing RH during drying (Fig. 3; Table 5). Furthermore, the standard errors tended to increase with decreasing values of *F*_v_/*F*_m_, particularly at prolonged recovery times. In general, humidity levels during drying did not have major effects on lichens in the absence of light, and the small humidity-dependent effects tended to decrease rapidly after 30 min of recovery. However, all RH treatments strongly impacted the lichens in the presence of light, as evidenced in the ANOVA by the highly significant light × humidity interaction (Table 5). The most severe drying treatment (0% RH) was particularly damaging in the presence of light. In all cyanolichens, the 35%, 55% and 75% RH levels gave clear, but relatively similar, levels of photoinhibition (Fig. 3); the largest contrast was between these humidity levels and the 0% RH treatment. Humidity-dependent differences in *F*_v_/*F*_m_ after the drying–light treatment were relatively constant during recovery in cyanolichens, whereas humidity-dependent contrasts in *F*_v_/*F*_m_ increased with recovery time in the two chlorolichens. This is one of the patterns that shaped the very strong interaction term between light and photobiont type (Table 5).

## Discussion

The old forest lichens studied were highly drying resistant in darkness with slightly greater damage in the two rarest species associated with the most humid climate (Table 3, Fig. 3). Doubling the drying period in darkness from 7 to 14 d did not decrease permanent PSII efficiency in *L. pulmonaria* (K. A. Solhaug, unpublished). In addition, the CO_2_ exchange in *L. pulmonaria* and the cyanolichen *Peltigera polydactyla* was normal after a 2-wk desiccation period over silica gel at low light/darkness (Kranner *et al.*, 2003). Light, however, strongly aggravated the damage caused by drying. The slight light-dependent differences in water content (Fig. 1) cannot explain the large contrast between damage after drying in light vs darkness (Fig. 3). As light, rather than drying alone, caused the recorded reduction in PSII efficiency, it is reasonable to believe that the susceptible partner is the photobiont. The reduction in PSII efficiency is probably a result of damage to PSII, as it is unlikely that a long-term nonphotochemical quenching caused by xanthophyll cycle pigments (Adams *et al.*, 2002) occurs in our thalli preconditioned for 48 h at low light whilst hydrated. Furthermore, conversion to zeaxanthin is unlikely during light exposure in dry conditions (Štepigová*et al.*, 2008). The cephalo- and chlorolichens with the strongest depression of PSII efficiency were also those in which damage in terms of bleaching occurred. Beyond reasonable doubt, we can conclude that light adversely affects the studied lichens during long dry periods (Table 3, Fig. 3). As studies in Swedish boreal forests have shown that *L. pulmonaria* can experience continuous periods in the dry state lasting for 700 h (Renhorn *et al.*, 1997), our 7-d exposure is ecologically relevant.

Old forest lichens are caught between a need to maximize light absorbance in active periods and to reduce light absorbance at the photobiont level during dry periods. Insufficient light in hydration periods often limits lichen growth in forests (Gauslaa *et al.*, 2006, 2007; Larsson *et al.*, 2012), as the light received during hydration periods is a strong factor promoting forest lichen growth (Dahlman & Palmqvist, 2003; Gaio-Oliveira *et al.*, 2004). Lichens use at least four strategies to reduce this conflict. Drying induces photoprotection by reducing light transmittance through the upper cortex to the underlying photobionts (Ertl, 1951; Büdel & Lange, 1994; Gauslaa & Solhaug, 2001), by curling lobes that shade the photobiont (Barták *et al.*, 2006), by functional disconnection of components of the photochemical apparatus (Sigfridsson, 1980; Bilger *et al.*, 1989) and by a mechanism that allows reaction centers of PSII to dissipate excess energy in the dry state (Heber *et al.*, 2006, 2010, 2011). For lichens of sun-exposed environments, photoprotective mechanisms are more efficient than for old forest lichens (Gauslaa & Solhaug, 1996). For old forest lichens, however, protective mechanisms cannot always prevent irreversible photoinhibition under field conditions (Gauslaa & Solhaug, 2000; Gauslaa *et al.*, 2001). The low level of intraspecific contrasts in light–drying tolerance between contrasting habitats for each of the studied Peltigerales species (Table 3) suggests that these lichens cannot easily escape damage by acclimation to local conditions. We believe that their association with old forests is at least partly shaped by their inability to cope with long-lasting light and drying in drier parts of their distribution areas. However, the widespread Parmeliaceae species *P. glauca* seems to be highly flexible with respect to habitat conditions (Fig. 2, Table 4).

The studied cyanolichens were much more resistant to extended dry and light periods than were experimental chloro- and cephalolichens. Based on the exceptionally high drying susceptibility of the cyanolichen *P. dissimilis* (Green *et al.*, 1991) and the strong association of epiphytic cyanolichens with rainy habitats (e.g. Marini *et al.*, 2011), the high resistance (Table 3, Fig. 3) was unexpected. *Nostoc*, the photobiont genus in our cyanolichens as well as in the drying-susceptible *P. dissimilis*, is known to be highly resistant to drying (Lüttge *et al.*, 1995; Lüttge, 2011). *Nostoc* has extracellular polysaccharides that are important for drying tolerance and rapid recovery of photosynthetic O_2_ evolution when rewetted (Tamaru *et al.*, 2005). These thick cyanobacterial sheaths store much water (Honegger *et al.*, 1996), slow down desiccation and possibly reduce drying damage. Different drying protocols between our study and the *P. dissimilis* study (Green *et al.*, 1991) may have caused the contrasting results. By placing hydrated thalli in a ventilated cuvette at very low RH, the drying rate was much faster in *P. dissimilis* than in our thalli that were first air dried at low light at 14°C and dried further in boxes with low constant RH. PSII of isolated green algal photobionts (*Trebouxia ericii*) recovers more slowly after rapid drying than after slower drying (Gasulla *et al.*, 2009).

**Fig. 4:**
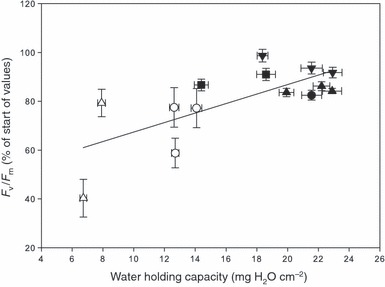
The relationship between thallus water holding capacity (WHC; from Gauslaa & Coxson, 2011) and permanent depression in *F*_v_/*F*_m_ measured in this study as a percentage of the start values after a 7-d combined drying and light exposure (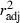 = 0.469; *P* = 0.005). Open symbols, the chlorolichens *Platismatia glauca* (triangles apex up) and *Lobaria pulmonaria* (circles). Closed symbols, *Pseudocyphellaria anomala* (squares), *Lobaria scrobiculata* (triangles apex down), *L. hallii* (triangles apex up) and *L. retigera* (circles). Each error bar (vertical as well as horizontal) represents ± 1SE (*n* = 39–40 for WHC and *n* = 19–20 for *F*_v_/*F*_m_) from each type of habitat (see Table 3).

To understand the strong association of epiphytic cyanolichens with humid climates, we may search for explanations other than low drying tolerance. Cyanolichens, unlike chloro- and cephalolichens, need liquid water to restore photosynthesis after drying (Lange *et al.*, 1986). In equilibrium with 90% RH, the cephalolichen *L. pulmonaria* assimilates carbon in the absence of liquid water, whereas the cyanolichen *L. scrobiculata* releases carbon, indicating some respiration, but no photosynthesis (Máguas *et al.*, 1995). Every specimen used in our study has known WHC (Gauslaa & Coxson, 2011). If we compare the mean *F*_v_/*F*_m_ values (expressed as a percentage of the start values) for all species–habitat combinations after the combined light–drying treatment (Table 2) with the corresponding WHC values reported in Gauslaa & Coxson (2011), there is a significant and positive interspecific regression (Fig. 4; 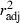 = 0.469; *P* = 0.005), but no significant intraspecific relationships (data not shown), between these two parameters. There is no overlap between the low WHC and *F*_v_/*F*_m_ values in green algal lichens with high values of both parameters in cyanolichens (Fig. 4). The significantly higher WHC in cyanolichens than in cephalo- and chlorolichens, driven by the higher specific thallus mass of these cyanolichens (Gauslaa & Coxson, 2011), is presumably a compensatory mechanism for their inability, as reported by Lange *et al.* (1986), to use humid air to activate photosynthesis. The studied chloro- and cephalolichens have thinner thalli than cyanolichens (Gauslaa & Coxson, 2011). Thin lichens take up humidity more rapidly than thick and compact lichens (Larson & Kershaw, 1976; Larson, 1981). Water-conserving cyanolichens often replace opportunistic chlorolichens with rapid water uptake and loss along a forested gradient from continental to oceanic climates. The frequent hydration–drying cycles in chlorolichens in continental sites with cool and humid nights allow regular photosynthetic activation and maintenance, which is not the case for cyanolichens. Cyanolichens depend on temporal rain and/or heavy dew fall and a large capacity to store water to maximize the length of rarer photosynthetic events. For example, an increase in WHC from 71 to 357 mg H_2_O cm^−2^ in *Degelia plumbea* prolongs its photosynthetic active period from 3 to 41 h (Gauslaa & Solhaug, 1998). The cyanolichen water economy requires a strong high-light resistance to handle the sometimes long periods between hydration periods, whereas chlorolichens may experience hydration every night in dry periods as a result of nocturnal cooling and the concurring rise in RH. In such a perspective, cyanolichens share a stress-tolerant strategy, whereas chlorolichens occupy the space between a competitive (*K*) strategy (best represented by the broad-lobed shading and shade-adapted cephalolichen *L. pulmonaria*) and a ruderal (*r*) strategy (various chlorolichens, such as the widespread *P. glauca*) in Grime’s (1977) triangle model of plant life history strategies (later applied for lichens by, for example, Rogers, 1990). At optimal hydration, the cyanolichen *L. scrobiculata* has rates of CO_2_ assimilation that are more than twice as high as those of the cephalolichen *L. pulmonaria*, but substantially lower relative growth rates under field conditions (Larsson *et al.*, 2012). The lower growth rate in *L. scrobiculata* is consistent with a stress-tolerant strategy.

Globally, cyanolichens tend to dominate the lower canopy of rain forests, whereas chlorolichens dominate the drier upper canopy (McCune, 1993; McCune *et al.*, 1997). A number of old forest cyanolichens, however, such as *L. scrobiculata*, can frequently occur in low biomass in open sites from continental inland areas. Such areas often experience a > 15°C drop in air temperature at nights during clear weather. When the temperature drops below the dew point, condensation replenishes the large WHC in cyanolichens, allowing early morning photosynthesis also in clear weather. This occurs in open stands from inland British Columbia (Y. Gauslaa, pers. obs. over a 2-wk period in early August 2011 showing hydration every clear morning), and may explain the strong growth response of *L. pulmonaria* to canopy gaps in old forests (Coxson & Stevenson, 2007a) and to forest edges (Coxson & Stevenson, 2007b), as well as the increasing growth response of *L. retigera* to increasing light (openness) at soft forest edges (Stevenson & Coxson, 2008). Cyanolichens of lichenized orders other than Peltigerales often grow on rocks or as soil crusts in arid climates (Büdel *et al.*, 1997; Belnap & Lange, 2005), where hydration by dew in clear nights is the main source of moisture and growth (Lange, 2001). The very high light–drying resistance of cyanolichens may be an ancient trait from before the land became shaded by plants. The epiphytic cyanolichen association with humid forests is probably not formed by a drying-susceptible *Nostoc* photobiont, but by the need for liquid water for the activation of photosynthesis and/or by a mycobiont being more susceptible than photobionts to drought-induced oxidative stress (Beckett *et al.*, 2003). The extreme sensitivity of *L. retigera* to edge effects (Stevenson & Coxson, 2008) may be shaped by its lower drying tolerance (Table 3).

The functional group cyanolichens includes cephalolichens (McCune, 1993). Most recent lichen studies applying the functional group concept have adopted this view. However, our study, Gauslaa & Coxson (2011) and photosynthetic studies all show that cephalolichens differ from cyanolichens in a number of functions and share important traits with chlorolichens. Field evidence shows that cephalolichens and cyanolichens tend to have only partly overlapping ecological niches. We thus follow Goward’s (2012) recommendations and distinguish between cephalo- and cyanolichens.

In conclusion, the old forest lichens studied were resistant to drying, whereas light exposure during drying could be highly detrimental. Cyanolichens, even rare old forest species associated with very humid climates, were substantially more resistant to combined drying–light treatments than were widespread chloro- and cephalolichens. The results lead to the following interpretation: cyanolichens depending on liquid water and large internal water storage require strong drying–light resistance to handle long periods between hydration events, whereas chlorolichens, which additionally and regularly activate photosynthesis by nocturnal humid air, do not need such high resistance.
